# Identification of protective peptides of *Fasciola hepatica*-derived cathepsin L1 (FhCL1) in vaccinated sheep by a linear B-cell epitope mapping approach

**DOI:** 10.1186/s13071-020-04260-6

**Published:** 2020-07-31

**Authors:** Leandro Buffoni, Laura Garza-Cuartero, Raúl Pérez-Caballero, Rafael Zafra, F. Javier Martínez-Moreno, Verónica Molina-Hernández, José Pérez, Álvaro Martínez-Moreno, Grace Mulcahy

**Affiliations:** 1 Animal Health Department (Parasitology and Parasitic Diseases), Faculty of Veterinary Medicine, University of Córdoba, Campus de Rabanales, Ctra. Madrid-Cádiz, km 396, 14014 Córdoba, Spain; 2 School of Veterinary Medicine, University College Dublin, Belfield, D4, Dublin, Ireland; 3 Laboratory of Malaria Immunology and Vaccinology, National Institute of Allergy and Infectious Diseases, National Institutes of Health, Bethesda, Maryland USA; 4 Anatomy and Comparative Pathology Department, Faculty of Veterinary Medicine, University of Córdoba, Campus de Rabanales, Ctra. Madrid-Cádiz, km 396, 14014 Córdoba, Spain; 5 Conway Institute, University College Dublin, Dublin, Ireland

**Keywords:** Epitope mapping, *Fasciola hepatica*, Vaccine, Sheep, Cathepsin L1

## Abstract

**Background:**

Fasciolosis is one of the most important parasitic diseases of livestock. The need for better control strategies gave rise to the identification of various vaccine candidates. The recombinant form of a member of the cysteine protease family, cathepsin L1 of *Fasciola hepatica* (FhCL1) has been a vaccine target for the past few decades since it has been shown to behave as an immunodominant antigen. However, when FhCL1 was used as vaccine, it has been observed to elicit significant protection in some trials, whereas no protection was provided in others.

**Methods:**

In order to improve vaccine development strategy, we conducted a linear B-cell epitope mapping of FhCL1 in sheep vaccinated with FhCL1, FhHDM, FhLAP and FhPrx plus Montanide and with significant reduction of the fluke burden, sheep vaccinated with FhCL1, FhHDM, FhLAP and FhPrx plus aluminium hydroxide and with non-significant reduction of the fluke burden, and in unvaccinated-infected sheep.

**Results:**

Our study showed that the pattern and dynamic of peptide recognition varied noticeably between both vaccinated groups, and that the regions 55–63 and 77–84, which are within the propeptide, and regions 102–114 and 265–273 of FhCL1 were specifically recognised only by vaccinated sheep with significant reduction of the fluke burden. In addition, these animals also showed significant production of specific IgG2, whereas a scarce non-significant production was observed in animals vaccinated with Aluminium hydroxide and no production was detected in infected control animals.

**Conclusions:**

We have identified 42 residues of FhCL1 that contributed to protective immunity against infection with *F. hepatica* in sheep. Our results provide indications in relation to key aspects of the immune response. Given the variable outcomes of vaccination trials conducted in ruminants to date, this study adds new insights to improve strategies of vaccine development.
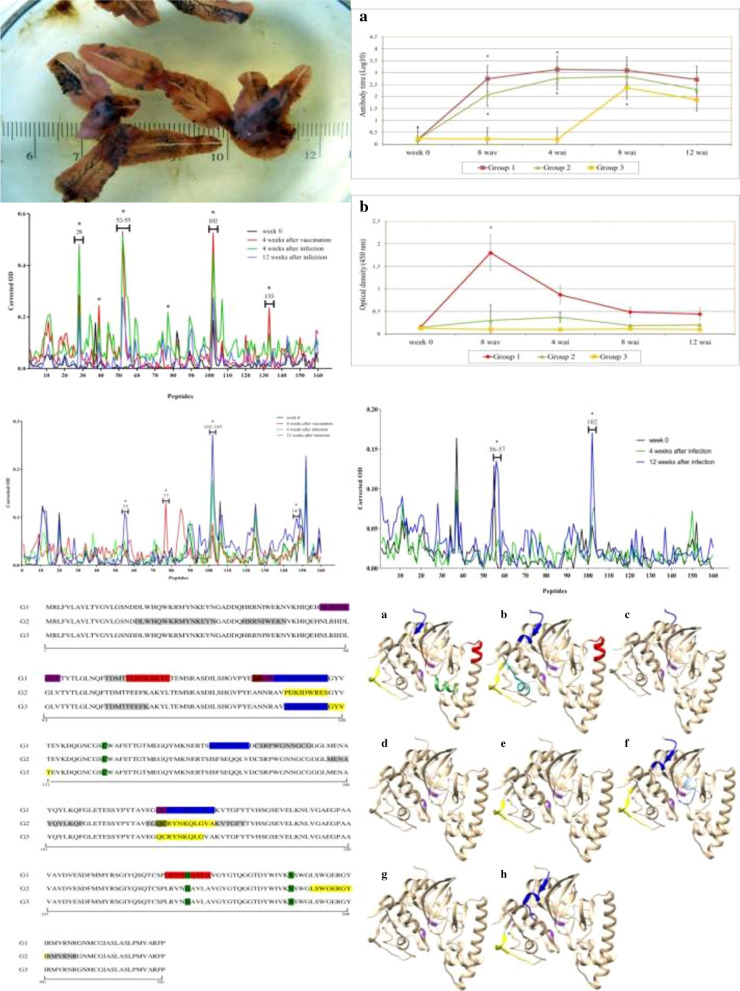

## Background

Fasciolosis is one of the most threatening parasitic diseases not only to agriculture but also to public health, as it is considered by the WHO as a re-emerging neglected tropical disease. It is associated with endemic disease in humans and it is believed that approximately 2.4 million people might be infected worldwide [1]. The urgent need for a vaccine to better control the disease gave rise to the identification of various potential vaccine candidates. These have been assessed over the past few decades with contrasting results in terms of their efficacy in producing protective immunity against infection. The difficulty in producing an effective vaccine is compounded by the immunomodulatory capacity of various *Fasciola hepatica* antigens, as has been extensively reported. Indeed, this immunomodulation includes suppression of dendritic cell maturation and function [2], activation of a suppressive dendritic cell population which weakens Th17 cells [3], induction of T cell anergy [4] and impairment of mast cells to drive Th1 responses [5].

Among the most promising vaccine candidates are the *Fasciola* cathepsins. For many years, these proteins have been proposed as a main target for vaccines as they are the predominant fraction of the excretory-secretory products of *F. hepatica* and play a dominant role during the invasive and migratory stages within the animal host and show a major capacity for immunoregulation [6–9]. Furthermore, they are immunodominant, and cathepsin-like proteases haven been used as an effective tool for serodiagnosis of fasciolosis in ruminants and humans [10, 11].

Numerous studies have reported the potential of these proteins in significantly reducing liver fluke burden, egg output or hepatic damage in cattle [12, 13], sheep [14, 15] and goats [16, 17], as vaccine components. However, there are also examples of vaccine trials where protection has not been achieved, so consistency is an issue to be overcome.

These discrepancies between vaccination trials have prompted a number of new approaches, including the identification of specific epitopes that may comprise part of a main immunodominant antigen such as cathepsins. These proteins have been shown to be highly present in the soluble secretome of the adult parasite [18] and within the exosome-like vesicles [19]. There is evidence that cathepsins play a key role during host-parasite interaction by causing degradation of the host extracellular matrix components which facilitates tissue migration [18], a strong humoral immune response [12–20] and modulation of the host immune response by various means [18, 21].

Differential epitope recognition by infected but not vaccinated, and vaccinated-infected animals with or without liver fluke burden reduction is a key tool in the identification of putative protective (and non-protective) epitopes. Although there is not much reported to date on epitope mapping of *F. hepatica*-derived antigens, some work has been conducted on other *F. hepatica* antigens including members of the saposin-like protein family [22] and glutathione S-transferase [23]. More recently, when epitopes of the MF6p/FhHDM-1 were mapped in vaccinated sheep, it was observed that the C-terminal region was more antigenic than the N-terminal region, and that production of specific antibodies followed a similar dynamic as for L-cathepsins [24]. With regard to members of the cysteine protease family, Harmsen et al. [25] identified protective peptides within the whole *F. hepatica* cathepsin L1 and L3 (FhCL1 and FhCL3) capable of inducing up to 63.6% of acquired resistance to infection in rats. Likewise, regions of FhCL1 were highly recognised by vaccinated-protected cattle during a B-cell epitope mapping study [26] and, what is more, when some synthetic peptide mimotopes of the FhCL1 were used as vaccine in sheep and goats, a significant level of protection between 35–79% was achieved [16, 27].

The aim of this study was to define linear B-cell epitopes recognised within the FhCL1 protein by antibodies in vaccinated and infected sheep, in order to specifically identify potential immunodominant peptides aimed at developing a feasible subunit vaccine against infection.

## Methods

### Animals

Thirty male Merino-breed sheep obtained from a liver fluke-free farm were used for the vaccination trial. Eighteen out of 30 were used for the epitope mapping study. Prior to beginning the study, all animals were confirmed to be free of liver fluke infection by coprological analyses and by ELISA for *F. hepatica* specific antibodies as previously described [13].

### Vaccine preparation

Two different vaccine formulations were prepared using recombinant forms of *F. hepatica* cathepsin L1 (FhCL1), *F. hepatica* helminth defence molecule (FhHDM), *F. hepatica* leucine aminopeptidase (FhLAP) and *F. hepatica* peroxiredoxin (FhPrx), which were combined with either Montanide or aluminium hydroxide (Alum) adjuvants. Each immunisation dose consisted of 100 µg of FhCL1, 100 µg of FhHDM, 100 µg of FhLAP and 100 µg of FhPrx plus 2 ml of adjuvant. FhCL1 was generated by expression of the cDNA in *Saccharomyces cerevisiae* as previously described [28]. Expression and purification of recombinant FhHDM were conducted in *Escherichia coli* [29]. FhLAP was obtained by cloning the cDNA in frame in BamHI and BglII sites of linearized pThio HisC *E. coli*, as previously described [30]. FhPrx was obtained by inserting the cDNA into the pPRO Ex HtA vector (Life Science Market - Gentaur Ltd. Hertfordshire, UK) and used to transform *E. coli* BL21-DE3 [31].

### Experimental design

For the vaccination trial, animals were randomly allocated into 3 groups of 10 animals each. Sheep from group 1 (G1) were vaccinated with FhCL1, FhLAP, FhHDM, FhPrx plus Montanide adjuvant, whereas animals from group 2 (G2) received FhCL1, FhLAP, FhHDM, FhPx combined with aluminium hydroxide adjuvant. Animals from group 3 (G3) were not vaccinated and remained as a positive control group. Vaccination was conducted subcutaneously, twice, with a 4-week-interval between doses. Eight weeks after the first vaccination, all animals were orally infected with a single dose of 150 *F. hepatica* metacercariae, South Gloucester strain (Ridgeway Research Ltd., St Briavels, UK) within a gelatine capsule using a bolus dosing gun. Fifteen weeks post-infection, sheep were euthanised by an intravenous injection of T61^®^ (Intervet, Barcelona, Spain).

Fluke burden was performed in a trial carried out with 10 animals per group, as mentioned above (our unpublished data). Six out of 10 animals from each group were used to carry out the epitope mapping study. Selection criteria of these animals were based on two parameters: the humoral immune response and the fluke burden. Prior to selection, all animals were analysed for production of antibodies, thus all selected animals were shown to produce specific anti-FhCL1 antibodies. Additionally, individual fluke burden (FB) was considered as follows; 2 out of 6 sheep per group harboured the highest and the lowest FB of each group, respectively, and the remaining 2 animals harboured an intermediate FB (see Additional file 1: Table S1).

### Parasitological study

To obtain the individual liver fluke burden in order to assess the development of protection against infection, during necropsy, all livers were collected and carefully dissected. The gallbladder and bile ducts were cut and opened and all flukes were counted and measured. Then, the liver was cut into small pieces and placed into warm water (40 °C) for 30 min to collect remaining flukes.

### Linear B-cell epitope mapping of FhCL1 by ELISA

For the epitope mapping study, blood samples were taken by jugular venepuncture before commencing the trial at day 0, 4 weeks after first vaccination (wav), 4 weeks after infection (wai, early stage of the infection) and 12 wai (late stage of the infection), plasma was obtained and stored at − 20 °C until use. It is worthwhile to mention that the other vaccine antigens (FhLAP, FhHDM and FhPrx) did not form part of the analysis.

The study was carried out according to the protocol of Garza-Cuartero et al. [26]. Briefly, a total of 160 overlapping peptides corresponding to the amino-acid sequence of FhCL1, each 9 amino acids in length with an overlap of 7 amino acids between successive peptides were synthesised (Mimotopes Pty. Ltd., Melbourne, Australia). Each of the peptides contained a biotin tag (see Additional file 1: Table S2) The FhCL1 reference sequence (MRLFVLAVLTVGVLGSNDDLWHQWKRMYNKEYNGADDQHRRNIWEKNVKHIQEHNLRHDLGLVTYTLGLNQFTDMTFEEFKAKYLTEMSRASDILSHGVPYEANNRAVPDKIDWRESGYVTEVKDQGNCGSCWAFSTTGTMEGQYMKNERTSISFSEQQLVDCSRPWGNNGCGGGLMENAYQYLKQFGLETESSYPYTAVEGQCRYNKQLGVAKVTGFYTVHSGSEVELKNLVGAEGPAAVAVDVESDFMMYRSGIYQSQTCSPLRVNHAVLAVGYGTQGGTDYWIVKNSWGLSWGERGYIRMVRNRGNMCGIASLASLPMVARFP) was obtained from UniProtKB: Q24940.

First, 96-well ELISA plates (Nunc. MaxiSorp^TM^; Thermo Fisher Scientific-Life Technologies Ltd, Paisley, UK) were coated overnight at 4 °C with 5 mg/ml of streptavidin (Sigma-Aldrich, Madrid, Spain). After 5 washes with phosphate buffered saline (PBS) 0.05% Tween 20, individual peptides were added in consecutive order to each well. Lyophilised peptides had been previously solubilised in 50% acetonitrile (Fisher Scientific, Loughborough, UK) in H_2_O (HyClone GE Healthcare Life Sciences, Little Chalfont, UK) and then diluted (1:20) in 0.1% sodium azide (Sigma-Aldrich) plus 0.1% in BSA (Sigma-Aldrich) in PBS. After dilution, biotinylated peptides were added (100 μl/well) at a final concentration of 50 mg/ml (in 0.1% sodium azide, 0.1% BSA in PBS). Plates were once again incubated overnight at 4 °C. After washing, plates were blocked using 2% BSA diluted in 0.05% PBS-Tween 20 (100 μl/well) for 90 min, washed, and 100 μl/well of plasma diluted at 1:50 in 2% BSA-PBS-Tween 20 was added, incubated for 90 min at room temperature (RT) with shaking. Plates were then washed and 100 μl/well of anti-sheep IgG-HRP (A3415; Sigma-Aldrich) diluted at 1:10,000 in 2% BSA-PBS-Tween was added and incubated for 90 min at RT. After washing, 100 μl/well of tetramethylbenzidine (TMB; Sigma-Aldrich) were added and incubated in the dark for 15 min at RT. The reaction was stopped by adding of 50 μl/well of 1 M sulphuric acid, and the absorbance was measured at 450 nm using a microplate photometer. Positive and negative plasma controls were included in each plate, as well as “blank wells” which consisted in wells which were not coated with peptides and wells coated with peptides but without plasma. The optical density of the blank wells (background) was subtracted from the wells containing peptides (and plasma samples) in each plate, hence final results are expressed as corrected OD (Additional file 1: Text S1).

### Detection of IgG1 and IgG2 anti-FhCL1 by ELISA

Briefly, 96-well ELISA plates were coated with 1 μg/ml of FhCL1 (100 μl/well), diluted in 0.05 M carbonate-bicarbonate buffer pH 9.6 and incubated at 37 °C overnight. After 5 washes with phosphate buffer saline (PBS) 0.05% Tween 20, plates were blocked with 100 μl/well of blocking buffer (BB) containing 1% BSA diluted in PBS and incubated at 37 °C for 30 min. To detect IgG1, wells were washed and 100 μl/well of plasma diluted in BB was added in duplicate and incubated for 30 min at 37 °C. Triple serial dilutions were performed to determine endpoint titre. To detect IgG2, 100 μl/well of plasma diluted at 1:10 in BB was added to each well in duplicate and incubated for 30 min at 37 °C. After washing, 100 μl/well of primary antibody diluted at 1:5000 (mouse anti-bovine IgG1 and anti-bovine IgG2 that cross-react with the ovine isotypes; 7500820–7500830, Cedi-Diagnostics, Lelystad, The Netherlands), in BB was added and incubated at 37 °C for 30 min. Then, wells were washed and anti-mouse IgG-HRP diluted at 1:500 was added at 37 °C for 30 min (STAR13B; Bio-Rad (formerly AbD-Serotec), Kidlington, UK). The plate was washed and 100 μl/well of TMB (Sigma-Aldrich) was added and incubated for 10 min at room temperature. The reaction was stopped by adding 100 μl/well of 1 M sulphuric acid and optical density was measured at 450 nm using a microplate photometer (Multiskan^TM^ FC; Thermo Fisher Scientific, Madrid, Spain). Results are shown as antibody titre − log10 - for IgG1, and as optical density for IgG2.

### Construction of the crystal 3-dimensional model of FhCL1

A 3D model of FhCL1 was built using the previous description of the crystal structure [8]. UniProtKB was used to obtain the models using the accession number Q24940 and RCSB PDB: 2O6X. All 3D diagrams were created using the programme UCSF CHIMERA 1.13.1 (Computer Graphics Laboratory, University of California, San Francisco, USA). The schematic representation of the 3D structure of FhCL1 is shown in Additional file 1: Figure S1. The active site of the protein is situated in the centre of the 3D molecule and formed by amino acids at positions Cys132, His269 and Asn289.

### Statistical analysis

Statistical analysis was performed with GraphPad Prism v6.0 (GraphPad Software, Inc., San Diego, CA, USA). To assess whether data distribution was parametric, the Kolmogorov-Smirnov test was used. For the analysis of production of specific IgG1–IgG2 against FhCL1 and for the epitope mapping study, a 2-way ANOVA and Bonferroni *post-hoc* test was used to compare differences between groups and time points. Statistical analysis of the recognised peptides was performed by comparing data of the different time points (4 wav, 4 and 12 wai) to data of week 0 (before commencing the trial). For fluke burden, a Kruskal-Wallis test with Dunn’s test was used. *P*-values of 0.05 or lower were considered statistically significant.

## Results

### Parasitological results

When data from vaccinated groups (G1-G2) were compared to that obtained in the control group (G3), a statistically significant protection of 37.3% for G1 and a non-significant protection of 13.8% for G2 was observed, in terms of reduction of the liver fluke burden.

### Antibody response to FhCL1

**Fig. 1:**
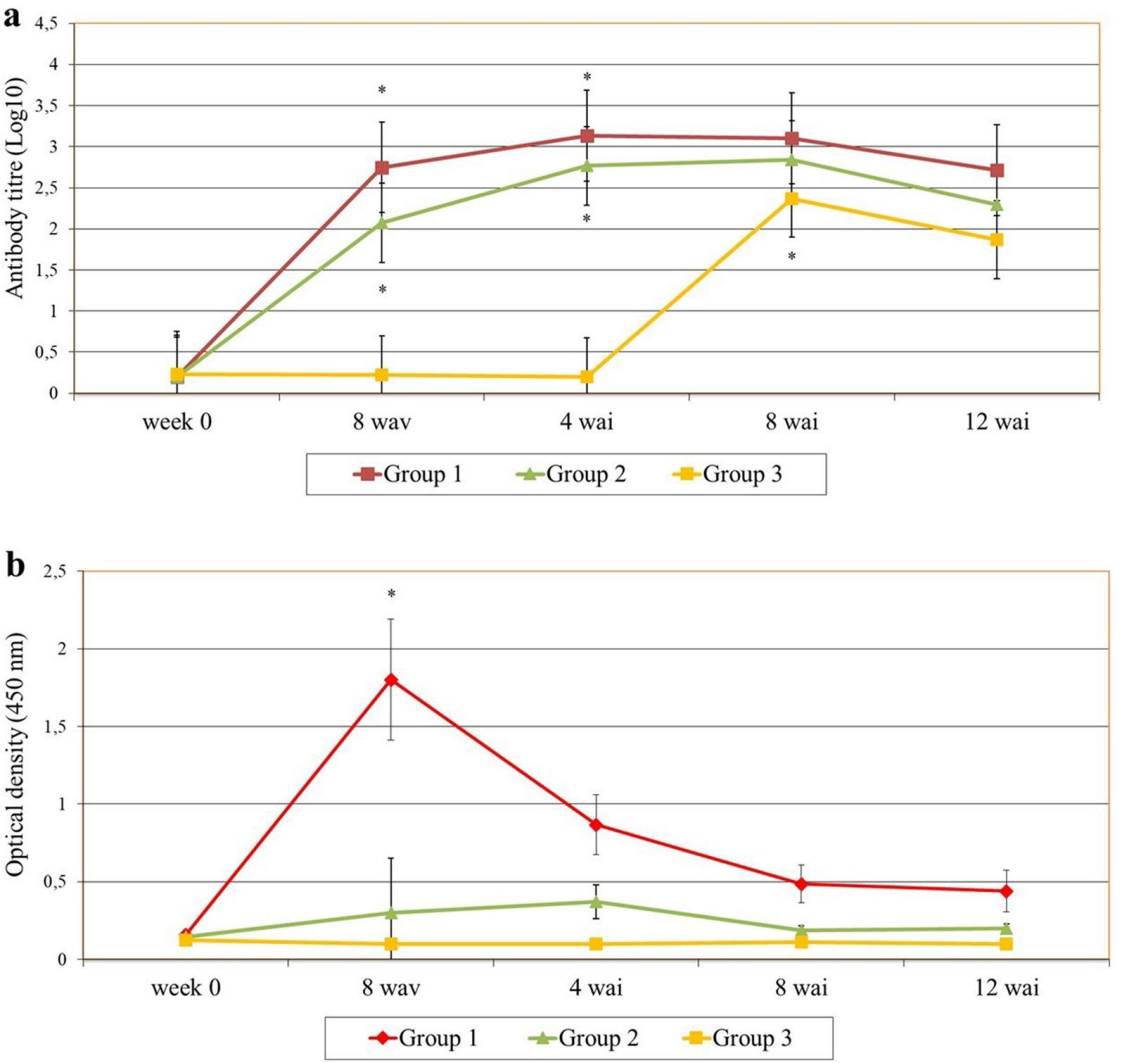
Levels of IgG1 **(a)** and IgG2 **(b)** that recognise FhCL1. Each line represents the mean values obtained at each time point, bars indicate standard error, asterisks indicate statistical significance (*P* < 0.001)

Production and dynamics of specific anti-FhCL1 IgG1 and IgG2 responses are presented in Fig. 1a, b, respectively. Although all vaccinated animals (G1-G2) developed a similar dynamic of IgG1 production during the trial, protected animals (G1) showed the highest levels at all time points. A sharp and significant increase of IgG1 was detected following vaccination (*P* < 0.001 for both G1–G2), reaching the maximum value 4 and 8 weeks after infection (wai) for group 1 and 2, respectively, and showing a gradual decrease from then onwards. Control animals (G3) showed significant production of IgG1 (*P* < 0.001) 8 weeks after infection (wai), though antibody levels were lower than those observed in vaccinated animals (Fig. 1a). In respect of IgG2, significant production was observed in animals from G1 (*P* < 0.001) at 8 wav. A lower non-significant production of IgG2 was detected in animals from G2. No production of IgG2 was observed in G3 (Fig. 1b).

### Epitope mapping of FhCL1 recognised by *F. hepatica* vaccinated and/or infected sheep

The level of specific recognition of peptides by each group at the different time points is summarised in Additional file 1: Table S3.

### Peptides recognised by vaccinated-Montanide sheep (G1)

**Fig. 2:**
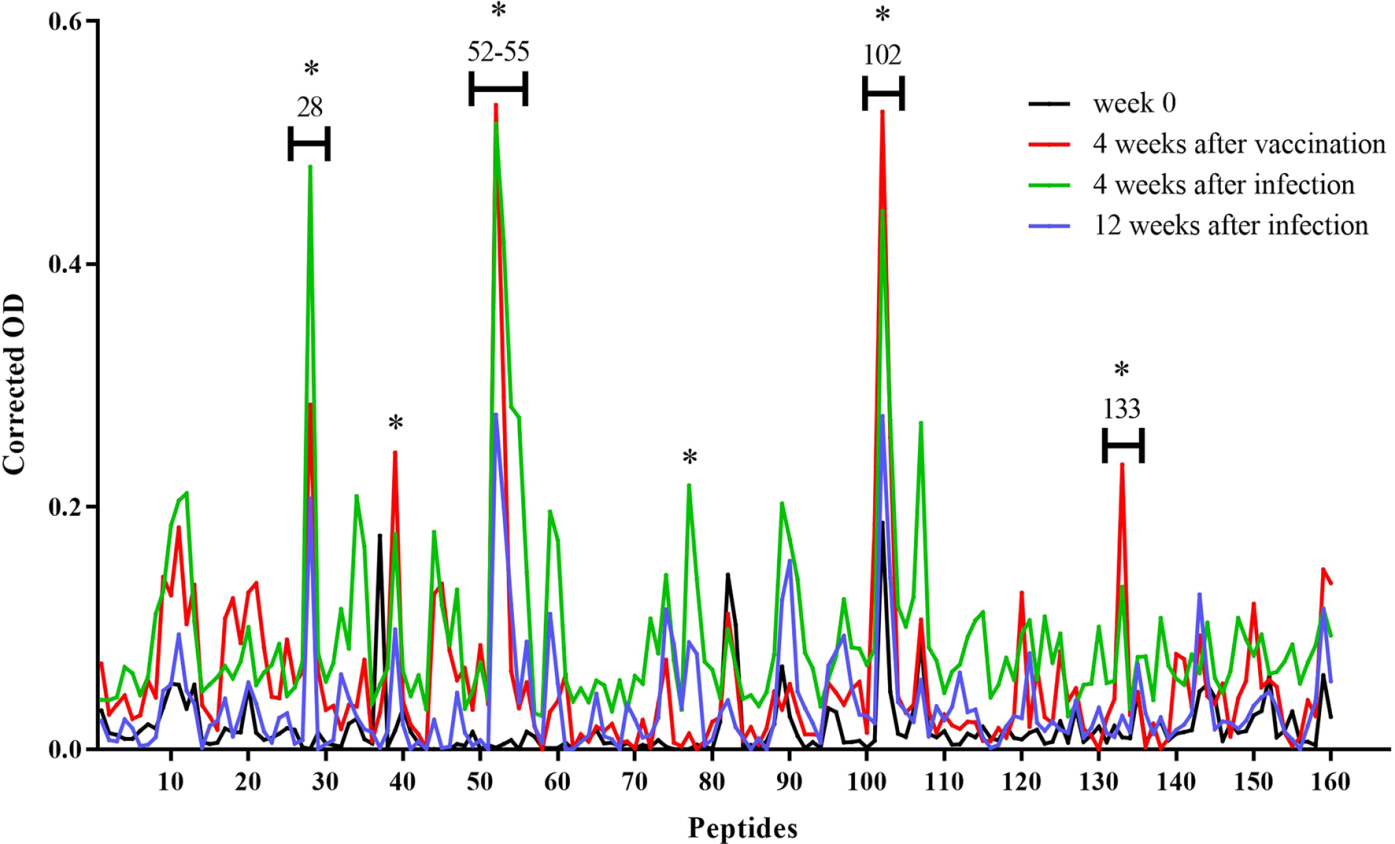
Dynamics of FhCL1-epitope mapping profile of animals from G1. Each line represents mean values per group (*n* = 6) at each time point and is expressed as corrected OD. Asterisks indicate statistical significance (for details see Additional file 1: Table S3)

The dynamics of peptide recognition is illustrated in Fig. 2. In the vaccinated-Montanide sheep, peptides 28 (*P* < 0.001), 39 (*P* = 0.021), 52–53 (*P* < 0.001), 102 (*P* < 0.001) and 133 (*P* = 0.032) were recognised after vaccination and before the infection (4 wav). After the experimental challenge, peptides 28 (*P* < 0.001), 52-53-54-55 (*P* < 0.001), 77 (*P* = 0.028), 102 (*P* = 0.0016) and 103 (*P* = 0.017) were recognised during the early stage of the infection (4 wai), whereas 52 (*P* < 0.001) was the only peptide recognised at the late stage of the infection (12 wai).

When comparing peptide recognition between the different time points, the highest binding was found for peptides 52–53 and 102 after vaccination and before infection, though a decreasing trend in reactivity was observed from that time point onwards. On the contrary, an increase in binding was observed for peptides 54 (*P* = 0.025) and 55 (*P* = 0.0059) at the early stage of the infection, when data from 4 wav and 4 wai was compared. This dynamic was similar for peptide 28, although no statistical difference was detected when data were compared.

### Peptides recognised by vaccinated-Alum sheep (G2)

**Fig. 3:**
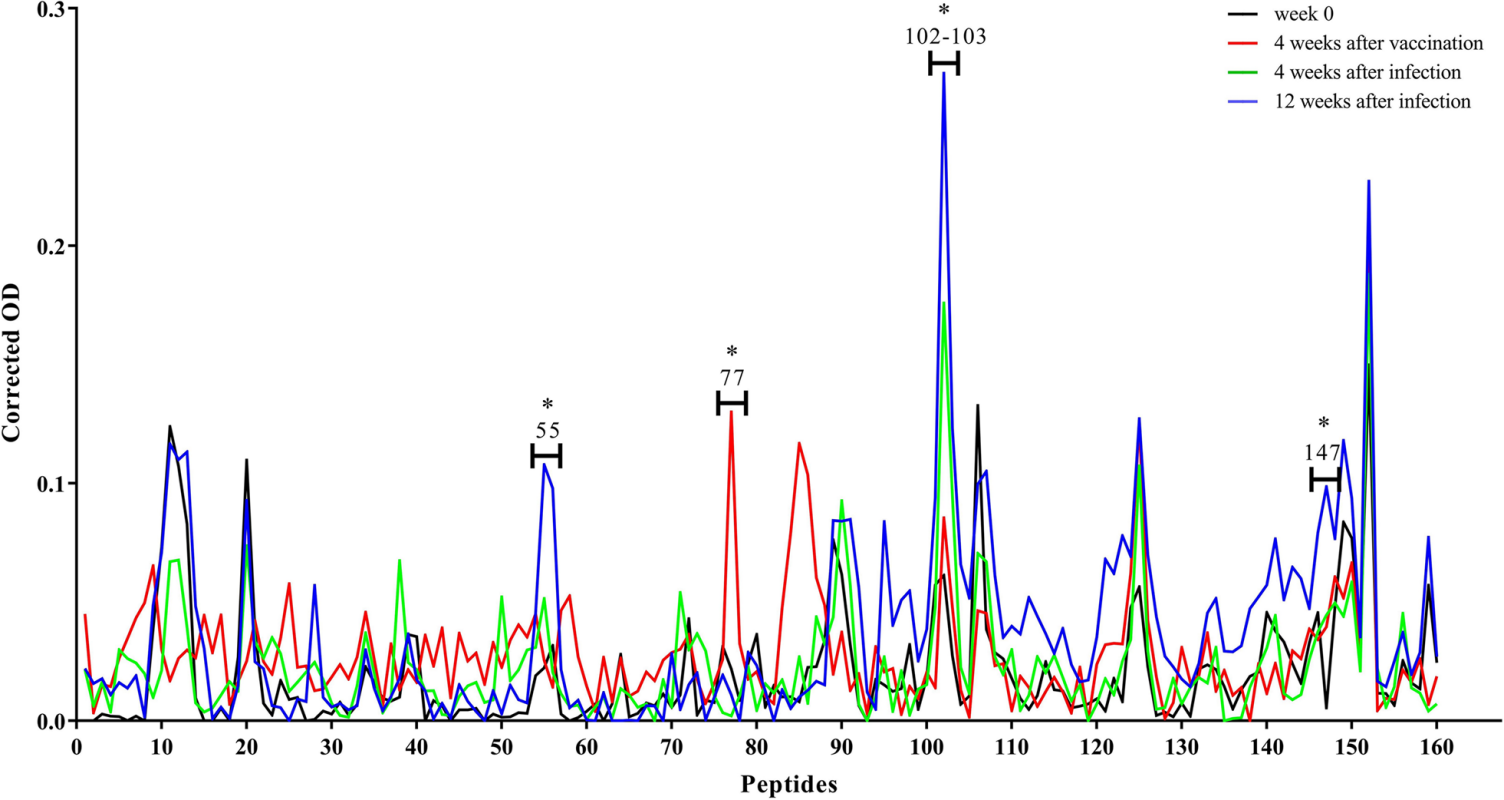
Dynamics of FhCL1-epitope mapping profile of animals from G2. Each line represents mean values per group (*n* = 6) at each time point and is expressed as corrected OD. Asterisks indicate statistical significance (for details see Additional file 1: Table S3)

Although peptides 77 and 85–87 were slightly recognised in one single animal after vaccination (4 wav), when mean data from G2 were analysed, no statistically significant recognition of these peptides was detected following vaccination. During the infection period, 102 was the only recognised peptide (*P* = 0.0012) at the early stage of the infection (4 wai), whereas peptides 55 (*P* = 0.0202), 102 (*P* < 0.001), 103 (*P* = 0.0029) and 147 (*P* = 0.0049) were specifically recognised at the late stage of the infection (12 wai). In contrast to what was observed in sheep from G1 with respect to peptide 102, an increasing trend in binding was detected after the experimental challenge (Fig. 3).

### Peptides recognised by unvaccinated and infected sheep (G3)

**Fig. 4:**
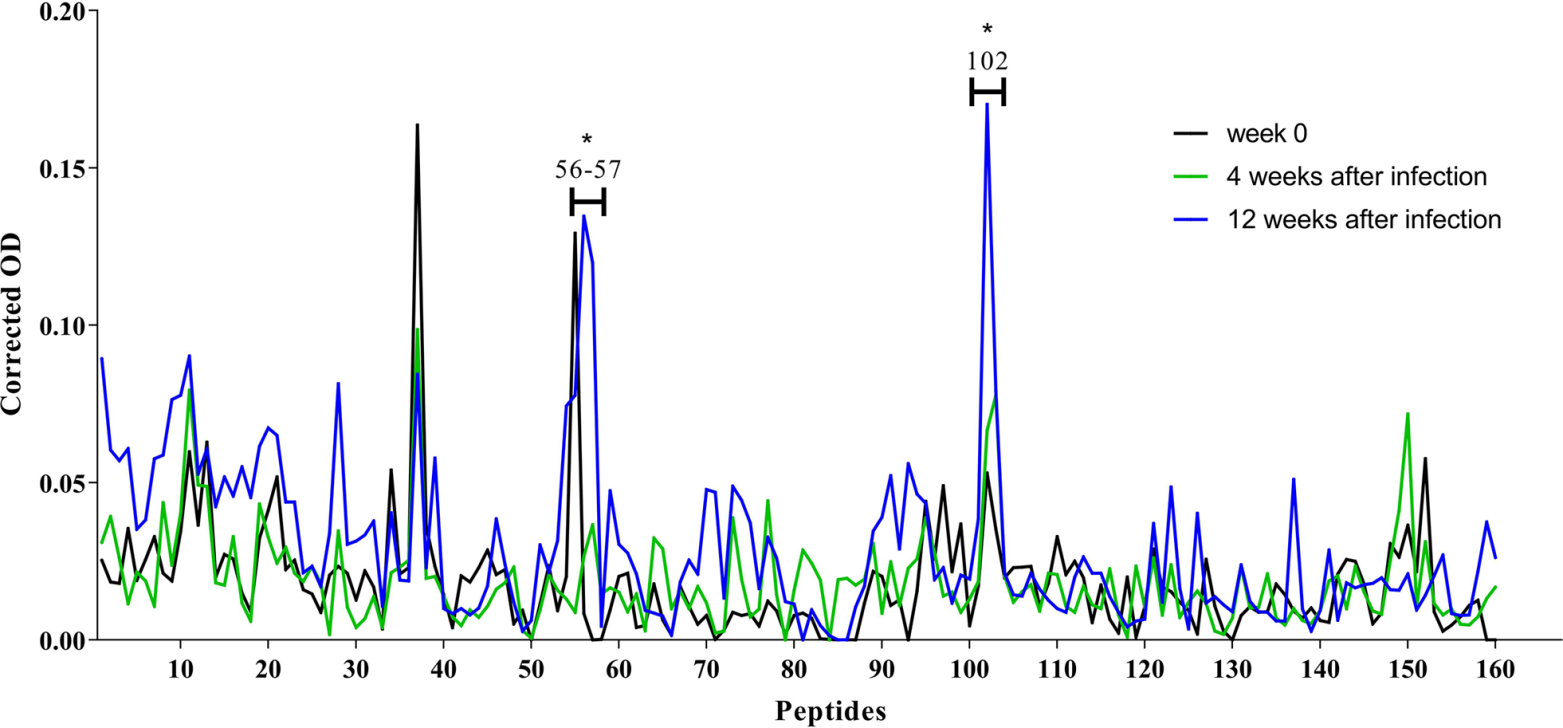
Dynamic of FhCL1-epitope mapping profile of animals from G3. Each line represents mean values per group (*n* = 6) at each time point and is expressed as corrected OD. Asterisks indicate statistical significance (for details see Additional file 1: Table S3)

In the infected control sheep, no peptides were specifically recognised at the early stage of the infection (4 wai). During the late stage of the infection (12 wai), peptides 56–57 and 102 were significantly recognised (*P* < 0.001). When data from the early a late stage of the infection were compared, a significant increase in the reactivity of peptides 56 (*P* < 0.001) and 102 (*P* = 0.0043) was detected at 12 wai (Fig. 4).

### Comparison of specifically recognised peptides between vaccinated (G1 and G2) and unvaccinated control animals (G3)

The dynamic of specific peptide recognition differed not only between vaccinated groups but also between vaccinated and unvaccinated animals. Peptides 28, 52-53-54 and 133 showed reactivity only in vaccinated-Montanide sheep, whereas peptides 56–57 were recognised exclusively in unvaccinated-infected sheep. Peptide 55 showed reactivity in all groups only after infection. Peptide 102 was recognised after infection in all groups, though in vaccinated animals it showed significant binding only in vaccinated-Montanide sheep (G1). Peptide 147 was only recognised in vaccinated-Alum group and at the late stage of the infection.

### Localisation of epitopes recognised by all groups in the linear sequence and in the 3D molecule of FhCL1

**Fig. 5:**
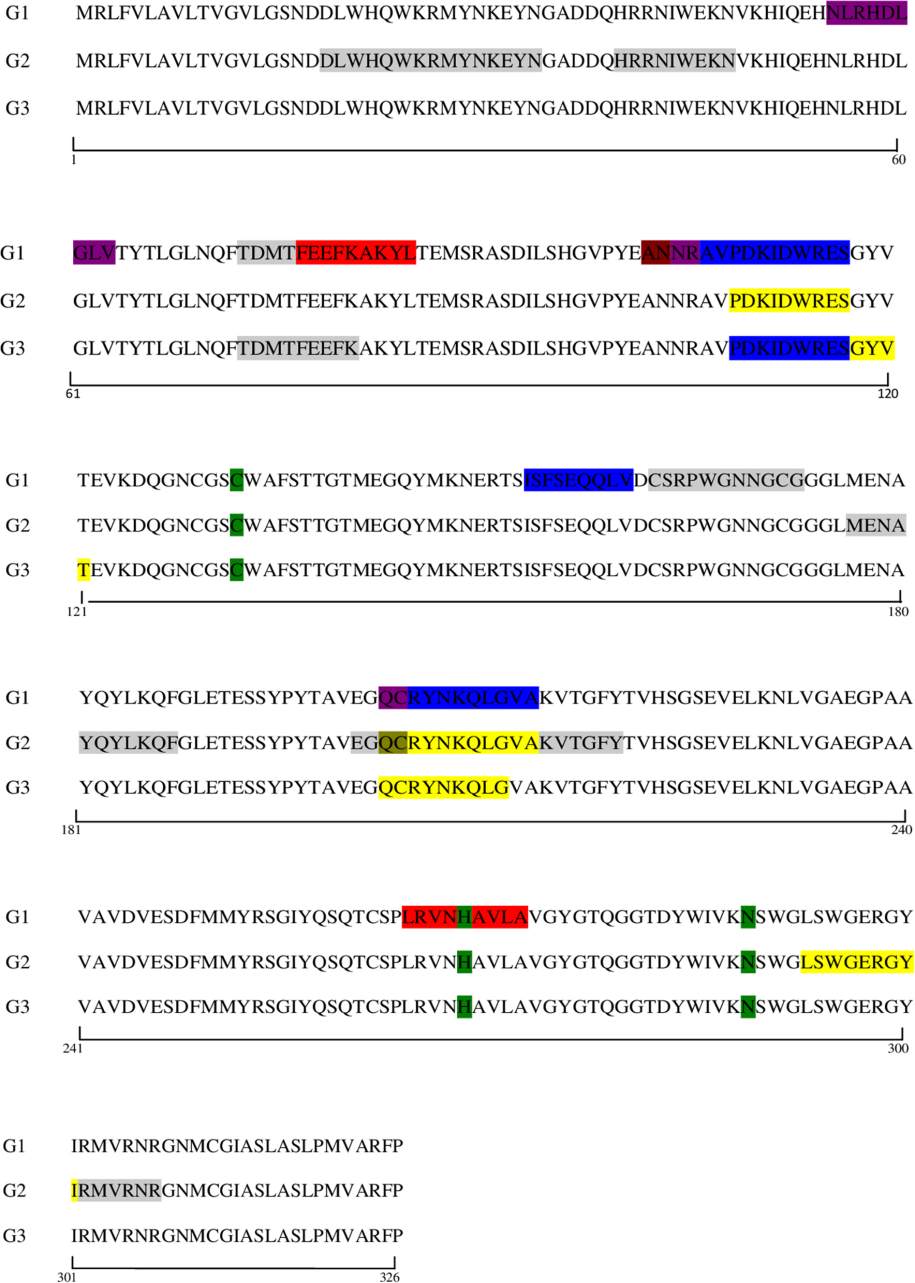
Localization of the peptides recognised in the linear FhCL1 sequence and comparison between groups and time points. *Key*: green, active sites of the protein; red, epitopes binding after vaccination and before infection (G1: peptide numbers 39, 133); blue, epitopes binding at the early stage of the infection (G1: peptide numbers 54-55-77-103); yellow, epitopes binding at the late stage of the infection (G2: peptide numbers 55-103-147; G3: peptide numbers 56-57-102); olive green, epitopes at early and late stage of the infection (G2: peptide number 102); purple, epitopes binding after vaccination and at the early infection (G1: peptide numbers 28-53-102); brown, epitopes binding at all time points (G1: peptide number 52); grey, non-specific binding of epitopes

**Fig. 6:**
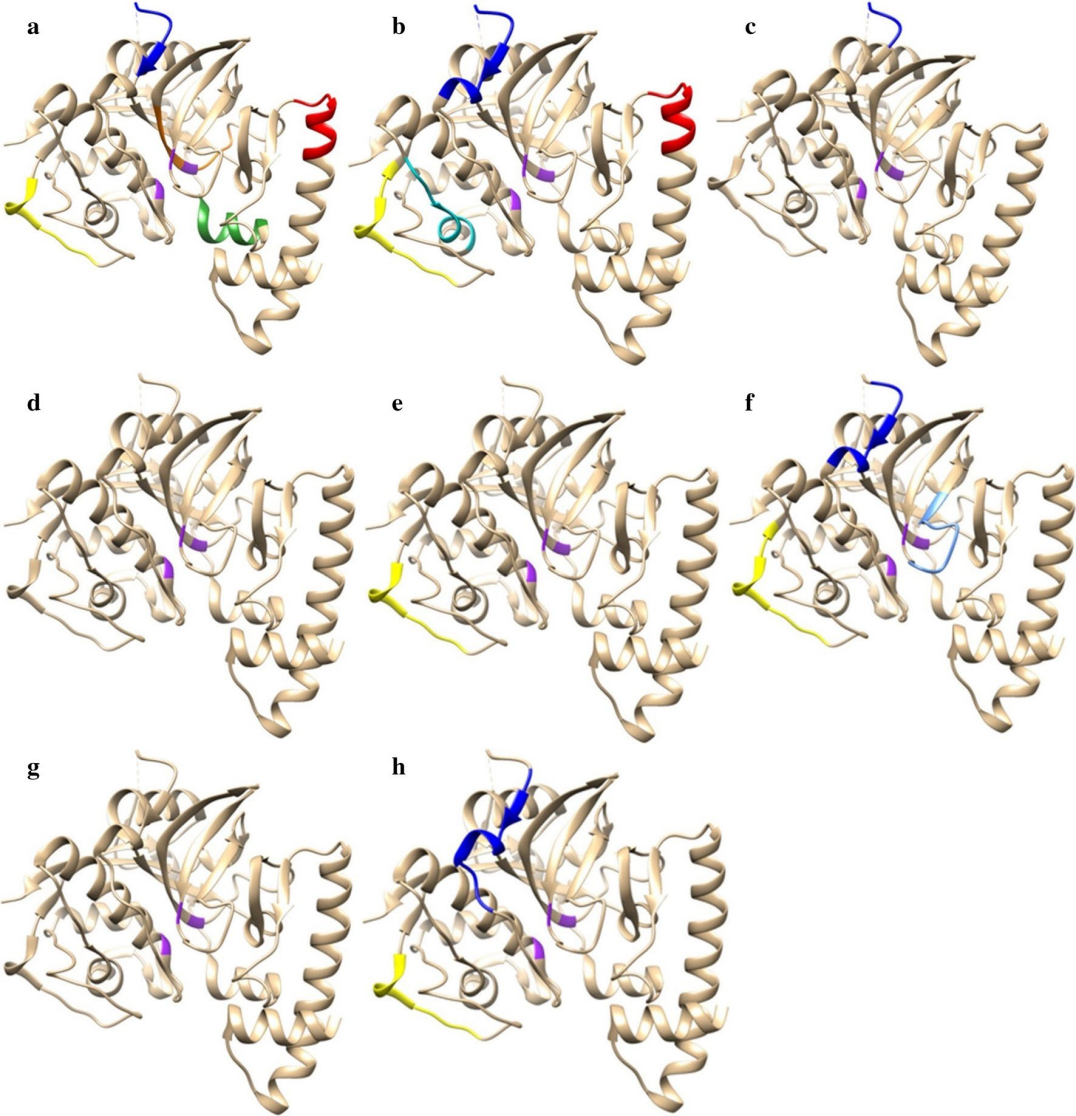
Epitope recognition and localisation in the 3D FhCL1 structure. **a** G1 (4 wav): red (aa 55–63, NLRHDLGLV); forest green (aa 77–84, FEEFKAKYL); blue (aa 102–112, ANNRAVPDKID); yellow (aa 203–211, QCRYNKQLG); orange (aa 265–273, LRVNHAVLA). **b** G1 (4 wai, early stage): red (aa 55–63, NLRHDLGLV); blue (aa 102–116, ANNRAVPDKIDWRES); cyan (aa 153–161, ISFSEQQLV); yellow (aa 203–213, QCRYNKQLGVA). **c** G1 (12 wai, late stage): blue (aa 102–110, ANNRAVPDK). **d** G2 (4 wav): no peptides were recognised at this time point. **e** G2 (4 wai, early stage): yellow (aa 203–211, QCRYNKQLG). **f** G2 (12 wai, late stage): blue (aa 108–116, PDKIDWRES); yellow (203–213, QCRYNKQLGVA); cornflower blue (293–301, LSWGERGYI). **g** G3 (4 wai, early stage): no peptides were recognised at this time point. **h** G3 (12 wai, late stage): blue (aa 110–120, KIDWRESGYVT); yellow (aa 203–211, QCRYNKQLG). Purple: active sites of the protein

After identifying specifically recognised peptides, the corresponding amino-acid regions of each overlapping peptide was localised in the linear sequence of FhCL1, in order to link peptide number with amino-acid positions and with amino-acid sequence within the whole FhCL1 molecule (Fig. 5). Simultaneously, highly identified epitopes were also mapped in the FhCL1 3D model (Fig. 6a-h).

The region localised on residue numbers 55–63 (NLRHDLGLV), which represents peptide 28, was recognised only in G1 after vaccination (Fig. 6a) and at the early stage of the infection (Fig. 6b). Peptide 55 situated in position 109–117, which corresponds to the sequence PDKIDWRES in the protein, was reactive only in vaccinated groups (G1 and G2) and after challenge though showing a contrasting dynamic during the early and late stage of the infection (Fig. 6a-f). This region of the protein forms part of the longer domain ANNRAVPDKIDWRES localised on residue numbers 105–117 (peptides 52-55) which was recognised in vaccinated-Montanide sheep (G1) at the early stage of the infection and was not further observed in the control group, and only at 12 wai in G2.

The sequence QCRYNKQLG (position 203–211, peptide 102) was consistently recognised by all groups, though showing a different dynamic during the trial. In the vaccinated-Montanide animals (G1) significant binding was detected right after vaccination (Fig. 6a), which was not observed in vaccinated-Alum sheep (G2), or during the early stage of the infection (Fig. 6b). Indeed, in G2 this region of the protein showed significant binding only after infection and displayed an increasing trend until the end of the study (Fig. 6d-f), which contrasts with the decreasing binding tendency observed in animals from G1. On the other hand, in the infected-control animals (G3), this sequence was only recognised at the late stage of the infection and showed the lowest level of reactivity when compared to G1 and G2 (Fig. 6g, h).

### Peptides recognised non-specifically

When analysing reactivity before beginning the trial, some peptides showed high binding against sheep antibody. In animals from G1, peptides 37, 82–83 and 102 showed non-specific reactivity in two different animals (Additional file 1: Table S4). In G2, up to 13 peptides were identified as non-specifically recognised, all of which were observed in one animal. In the positive control group (G3), peptides 37 and 55 showed reactivity in one animal.

## Discussion

Over the past number of years, considerable progress has been made in the identification of molecules for vaccine development against infection with *F. hepatica* but unfortunately, inconsistent results have been obtained in terms of vaccine efficacy.

FhCL1 is one of the dominant molecules in the secretome of the mature form of *F. hepatica* [18] and plays an important role during host-parasite interaction, with various reports showing it contributes to the immunomodulatory strategy of the parasite [32–34]. In this study, we have analysed the dynamic of the antibody response to FhCL1 and we have conducted a linear B-cell epitope identification of the FhCL1 by plasma obtained from vaccinated sheep with and without significant reduction of the fluke burden, and from unvaccinated-infected animals.

First, we observed a significant production of specific anti-FhCL1 IgG1 after vaccination in both vaccinated groups and also after infection in the infected-control sheep, as expected and observed in previous studies [12, 17, 20, 26]. In addition, when data were compared, IgG1 level was highest throughout the trial in the vaccinated-Montanide group. These results are in line with those observed in previous studies conducted in cattle and goats vaccinated with FhCL1 and naturally and experimentally challenged with *F. hepatica* [12, 20], in which a similar level of specific IgG1 production was observed. We also found that specific IgG2 production correlated with vaccine-induced protection. This finding has also been reported in cattle after FhCL1 vaccination, hence, as it has been formerly hypothesised [12, 26, 35, 36], an elevated level of IgG2 might be considered as a feasible indicator of protection.

Secondly, we analysed the epitope mapping study of the FhCL1 and we observed striking differences in the pattern of specific peptide recognition not only between vaccinated groups but also between vaccinated and only-infected animals. The region spanning amino acids 102–114 (ANNRAVPDKIDWR) was recognised exclusively in the vaccinated-Montanide group, whereas the region 108–116 (PDKIDWRES) was identified in both vaccinated groups but not in the control group. Moreover, we observed that the overlapped sequence ANNRAVPDK (region 102–110) reacted solely in the vaccinated-Montanide sheep after vaccination and during the early and late stage of the infection. It is worth noting that this region and adjacent amino acids 102–112 (ANNRAVPDKID) showed a decreasing recognition throughout the trial and that the overlapping region 104–112 (NRAVPDKID) was no longer detected at the chronic stage of the infection (12 wai) in G1. This finding aligns with those reported in a previous study conducted in rats, in which vaccination with a synthetic peptide of FhCL1 comprising the region 104–122 (sequence NRAVPDKIDWRESGYVTE, in part overlapping the region described above) resulted in a significant 40% reduction of the fluke burden [25]. On the contrary, this region was not recognised in cattle, either in partially protected animals vaccinated with FhLC1 or in vaccinated-unprotected or unvaccinated-infected cattle [26].

Another peptide that was consistently recognised in our study is situated at the region spanning amino acids 203–211 (QCRYNKQLG). This region, which is positioned at the periphery of the 3D molecule and therefore, easily exposed to recognition of antibodies, was identified in all groups though displaying a distinct dynamic throughout the trial. Curiously, this sequence was reactive four weeks after vaccination and at the early stage of the infection in vaccinated-Montanide group, whereas no post-vaccination reaction was observed in the vaccinated-Aluminium hydroxide group. What is more surprising, when data from vaccinated groups were compared, is that no peptide recognition was detected following immunisation in sheep vaccinated with four antigens plus alum in comparison to the group where Montanide was used. In addition, IgG1 production was higher with Montanide as adjuvant, whereas production of IgG2 in animals with alum was scarce. These findings in antigenic recognition between groups immunised with different adjuvants, reflect a possible role of these in the development of protective immune responses. In a recent study, it was claimed that alum elicited a downregulation in cytokines and cytokine receptors in ovine-derived PBMCs [37]. Although alum-based adjuvants have been used for many decades, their mechanism of action still remains elusive, aspects that have been recently reviewed by Ghimire et al. [38]. Therefore, in our study it was clearly shown that both adjuvants significantly influenced the B-cell immune response in a different way and, hence, the observation of the lack of reaction to specific epitopes after vaccination in sheep immunised with aluminium-based vaccine. However, no adjuvant-control groups have been included in our study, which might have helped to elucidate the role of both adjuvants.

Additionally, we also observed that the sequences NLRHDLGLV (aa 55–63) and FEEFKAKYL (aa 77–84), which are within the pro-peptide, and the sequence LRVNHAVLA (aa 265–273) were specifically recognised after vaccination only by G1. The latter belongs to a larger region (aa 260–279, QTCSPLRVNHAVLAVGYGTQ) shown to elicit elevated levels of INF-γ in a murine model [39]. However, these regions showed no specific reaction in protected sheep immunised with mimotopes of FhCL1 lacking adjuvant [27]. As in *F. hepatica*-infected and protected cattle [26] we also observed specific antibodies to the pro-peptide of FhCL1 in sheep with significant reduction of the fluke burden. Whether these antibodies would be predicted to be protective in ruminants as it was previously observed in rats [25] is yet unclear and needs to be clarified.

What is particularly noteworthy is that our findings contrast with those reported recently in vaccinated cattle, in which protective linear B-cell epitopes of FhCL1 were identified by following the same epitope mapping approach. That is, epitopes recognised in vaccinated sheep in this study were not identified in vaccinated cattle with FhCL1, and *vice versa* [26]. This could perhaps be due to key differences on the vaccination protocol or time points analysed between trials, or on the different cellular or molecular mechanisms involved in the immune response pathways between sheep and cattle. Indeed, marked discrepancies in epitope recognition were also observed between animals sharing the same pathogen. In ruminants infected with bovine leukemia virus, it was observed that sheep and cattle displayed a different range of epitope specificity, which means that antibodies can be targeted to a different region of the same antigen in both animal species [40]. Understanding whether differential epitope recognition caused by divergences on Ag presentation pathway is host mediated, will contribute to better knowledge of the biology of the immune response during liver fluke infection. Contrasting data between cattle and sheep infected with *F. hepatica* have also been observed in terms of the cellular immune response, in particular with regard to cell subsets involved in antigen presentation process. For instance, γδTCR cells which display various functional responses (including antigen presentation) [41, 42] were shown to be depleted during the early stage of the infection in cattle [43] in contrast to what has been detected in sheep [44]. This cell subset is not MHC restricted and can recognise peptides *via* a mechanism that requires direct cell-cell contact with an antigen presenting cell and signalling through the γδTC [45–47]. In addition, it was recently reported in two transcriptomic studies conducted in cattle and sheep infected with *F. hepatica* that major differences exist in the expressed genes related to immune response, and that the proportion of differentially expressed genes (DEGs) observed between acute and chronic stages of the infection was responsible for up to more than 70% of the total DEGs at the acute stage in sheep, whereas this percentage decreased to about 5% in cattle [48, 49].

Furthermore, other noticeable similarities and differences between our findings and those reported in cattle in terms of epitope mapping [26], include the overall recognition and localisation in the linear and within the 3D model of FhCL1 in protected animals. Although the main recognised protective peptides in both trials are localised at the periphery of the 3D model and face outwards from the body of the molecule, it seems that an earlier specific recognition of peptides occurs in sheep, in contrast to the later recognition in cattle.

On the other hand, we noticed that non-specific epitope recognition occurred in some animals. Nine regions comprising up to 104 different residues were detected. Among them, the region 203–211, which has shown a constant reaction in all groups as commented above, showed non-specific recognition before vaccination in two animals from vaccinated groups. Similarly, the region 108–116 showed non-specific binding by antibodies before infection in one animal from the unvaccinated infected group. These results differ from those reported in cattle in terms of non-specific epitope binding [26]. Since all animals were managed under identical conditions and no coexisting disease was observed during the trial, we therefore suggest the non-specific reactivity might be due to the presence of an undetected infection by other pathogens sharing similar epitope conformation with FhCL1 that might have led some peptides to cross-react with antibodies.

To sum up, this study shows different antigenic recognition in sheep in relation to other ruminant species, a fact that could be justified by specific differences in the development of the immune response or aspects related to the experimental protocols used in each case.

## Conclusions

We have identified specific regions of FhCL1 comprising up to 42 residues, 23 of which were situated within the pro-peptide, that contributed to protective immunity against infection with *F. hepatica* in sheep. The induction of certain humoral immune responses such as those related to the IgG2 isotype production when Montanide was used as adjuvant, determines the recognition of certain overlapping peptides of FhCL1, different from those observed in the other experimental groups. Given the divergent outcomes of vaccination trials conducted in ruminants either with single or multi-antigen-based vaccines, identification of protective peptides by mapping epitopes of immunodominant *F. hepatica* antigens adds new insights to improve strategies of vaccine development.

## Supplementary information

**Additional file 1: Table S1.** Individual fluke burden. **Table S2.** overlapping peptides of FhCL1. **Text S1.** Formula corrected OD. **Figure S1.** 3D model of FhCL1. **Table S3.** Level of statistical significance of peptides recognised in each group. **Table S4.** Non-specifically recognised peptides.

## Data Availability

Data supporting the conclusions of this study are included within the article and its additional file. Raw data are available from the corresponding author upon request.

## Abbreviations

WHO: World Health Organisation
FhCL1: recombinant *Fasciola hepatica* cathepsin L1
FhHDM: recombinant *Fasciola hepatica* helminth defence molecule
FhLAP: recombinant *Fasciola hepatica* leucine aminopeptidase
FhPrx: recombinant *Fasciola hepatica* peroxiredoxin
wav: weeks after vaccination
wai: weeks after infection
alum: aluminium hydroxide
