# Supplementary material for: Identification of protective peptides of Fasciola hepatica-derived cathepsin L1 (FhCL1) in vaccinated sheep by a linear B-cell epitope mapping approach
Source: Parasit Vectors. 2020 Jul 31;13:390. doi: 10.1186/s13071-020-04260-6 (PMC7393625; doi:10.1186/s13071-020-04260-6)
Supplement: Supplementary file 1 — Additional file 1: Table S1. Individual fluke burden. Table S2. overlapping peptides of FhCL1. Text S1. Formula corrected OD. Figure S1. 3D model of FhCL1. Table S3. Level of statistical significance of peptides recognised in each group. Table S4. Non-specifically recognised peptides. [file 13071_2020_4260_MOESM1_ESM.doc]

**Additional file 1: Table S1.** Individual fluke burden of animals of the epitope mapping study. The mean liver fluke burden per group derived from a vaccination trial in which each group was formed by ten animals (unpublished data). Six out of ten animals of each group were used to carry out the epitope mapping study.

| **Animals** | **Group 1** | **Group 2** | **Group 3** |
| --- | --- | --- | --- |
| **1** | 19 | 45 | 44 |
| **2** | 36 | 49 | 56 |
| **3** | 42 | 53 | 73 |
| **4** | 45 | 56 | 75 |
| **5** | 53 | 64 | 86 |
| **6** | 56 | 76 | 86 |

**Additional file 1: Table S2.** Overlapping peptides of FhCL1. To connect to biotin, the sequence SGSG-peptide was added to each peptide (red).

| **Pep No** | **NTerm** | **Sequence** | **Pep No** | **NTerm** | **Sequence** | **Pep No** | **NTerm** | **Sequence** | **Pep No** | **NTerm** | **Sequence** |
| --- | --- | --- | --- | --- | --- | --- | --- | --- | --- | --- | --- |
| **1** | H- | MRLFVLAVLGSG | **41** | Biotin- | SGSGKAKYLTEMS | **81** | Biotin- | SGSGVDCSRPWGN | **121** | Biotin- | SGSGVAVDVESDF |
| **2** | Biotin- | SGSGLFVLAVLTV | **42** | Biotin- | SGSGKYLTEMSRA | **82** | Biotin- | SGSGCSRPWGNNG | **122** | Biotin- | SGSGVDVESDFMM |
| **3** | Biotin- | SGSGVLAVLTVGV | **43** | Biotin- | SGSGLTEMSRASD | **83** | Biotin- | SGSGRPWGNNGCG | **123** | Biotin- | SGSGVESDFMMYR |
| **4** | Biotin- | SGSGAVLTVGVLG | **44** | Biotin- | SGSGEMSRASDIL | **84** | Biotin- | SGSGWGNNGCGGG | **124** | Biotin- | SGSGSDFMMYRSG |
| **5** | Biotin- | SGSGLTVGVLGSN | **45** | Biotin- | SGSGSRASDILSH | **85** | Biotin- | SGSGNNGCGGGLM | **125** | Biotin- | SGSGFMMYRSGIY |
| **6** | Biotin- | SGSGVGVLGSNDD | **46** | Biotin- | SGSGASDILSHGV | **86** | Biotin- | SGSGGCGGGLMEN | **126** | Biotin- | SGSGMYRSGIYQS |
| **7** | Biotin- | SGSGVLGSNDDLW | **47** | Biotin- | SGSGDILSHGVPY | **87** | Biotin- | SGSGGGGLMENAY | **127** | Biotin- | SGSGRSGIYQSQT |
| **8** | Biotin- | SGSGGSNDDLWHQ | **48** | Biotin- | SGSGLSHGVPYEA | **88** | Biotin- | SGSGGLMENAYQY | **128** | Biotin- | SGSGGIYQSQTCS |
| **9** | Biotin- | SGSGNDDLWHQWK | **49** | Biotin- | SGSGHGVPYEANN | **89** | Biotin- | SGSGMENAYQYLK | **129** | Biotin- | SGSGYQSQTCSPL |
| **10** | Biotin- | SGSGDLWHQWKRM | **50** | Biotin- | SGSGVPYEANNRA | **90** | Biotin- | SGSGNAYQYLKQF | **130** | Biotin- | SGSGSQTCSPLRV |
| **11** | Biotin- | SGSGWHQWKRMYN | **51** | Biotin- | SGSGYEANNRAVP | **91** | Biotin- | SGSGYQYLKQFGL | **131** | Biotin- | SGSGTCSPLRVNH |
| **12** | Biotin- | SGSGQWKRMYNKE | **52** | Biotin- | SGSGANNRAVPDK | **92** | Biotin- | SGSGYLKQFGLET | **132** | Biotin- | SGSGSPLRVNHAV |
| **13** | Biotin- | SGSGKRMYNKEYN | **53** | Biotin- | SGSGNRAVPDKID | **93** | Biotin- | SGSGKQFGLETES | **133** | Biotin- | SGSGLRVNHAVLA |
| **14** | Biotin- | SGSGMYNKEYNGA | **54** | Biotin- | SGSGAVPDKIDWR | **94** | Biotin- | SGSGFGLETESSY | **134** | Biotin- | SGSGVNHAVLAVG |
| **15** | Biotin- | SGSGNKEYNGADD | **55** | Biotin- | SGSGPDKIDWRES | **95** | Biotin- | SGSGLETESSYPY | **135** | Biotin- | SGSGHAVLAVGYG |
| **16** | Biotin- | SGSGEYNGADDQH | **56** | Biotin- | SGSGKIDWRESGY | **96** | Biotin- | SGSGTESSYPYTA | **136** | Biotin- | SGSGVLAVGYGTQ |
| **17** | Biotin- | SGSGNGADDQHRR | **57** | Biotin- | SGSGDWRESGYVT | **97** | Biotin- | SGSGSSYPYTAVE | **137** | Biotin- | SGSGAVGYGTQGG |
| **18** | Biotin- | SGSGADDQHRRNI | **58** | Biotin- | SGSGRESGYVTEV | **98** | Biotin- | SGSGYPYTAVEGQ | **138** | Biotin- | SGSGGYGTQGGTD |
| **19** | Biotin- | SGSGDQHRRNIWE | **59** | Biotin- | SGSGSGYVTEVKD | **99** | Biotin- | SGSGYTAVEGQCR | **139** | Biotin- | SGSGGTQGGTDYW |
| **20** | Biotin- | SGSGHRRNIWEKN | **60** | Biotin- | SGSGYVTEVKDQG | **100** | Biotin- | SGSGAVEGQCRYN | **140** | Biotin- | SGSGQGGTDYWIV |
| **21** | Biotin- | SGSGRNIWEKNVK | **61** | Biotin- | SGSGTEVKDQGNC | **101** | Biotin- | SGSGEGQCRYNKQ | **141** | Biotin- | SGSGGTDYWIVKN |
| **22** | Biotin- | SGSGIWEKNVKHI | **62** | Biotin- | SGSGVKDQGNCGS | **102** | Biotin- | SGSGQCRYNKQLG | **142** | Biotin- | SGSGDYWIVKNSW |
| **23** | Biotin- | SGSGEKNVKHIQE | **63** | Biotin- | SGSGDQGNCGSCW | **103** | Biotin- | SGSGRYNKQLGVA | **143** | Biotin- | SGSGWIVKNSWGL |
| **24** | Biotin- | SGSGNVKHIQEHN | **64** | Biotin- | SGSGGNCGSCWAF | **104** | Biotin- | SGSGNKQLGVAKV | **144** | Biotin- | SGSGVKNSWGLSW |
| **25** | Biotin- | SGSGKHIQEHNLR | **65** | Biotin- | SGSGCGSCWAFST | **105** | Biotin- | SGSGQLGVAKVTG | **145** | Biotin- | SGSGNSWGLSWGE |
| **26** | Biotin- | SGSGIQEHNLRHD | **66** | Biotin- | SGSGSCWAFSTTG | **106** | Biotin- | SGSGGVAKVTGFY | **146** | Biotin- | SGSGWGLSWGERG |
| **27** | Biotin- | SGSGEHNLRHDLG | **67** | Biotin- | SGSGWAFSTTGTM | **107** | Biotin- | SGSGAKVTGFYTV | **147** | Biotin- | SGSGLSWGERGYI |
| **28** | Biotin- | SGSGNLRHDLGLV | **68** | Biotin- | SGSGFSTTGTMEG | **108** | Biotin- | SGSGVTGFYTVHS | **148** | Biotin- | SGSGWGERGYIRM |
| **29** | Biotin- | SGSGRHDLGLVTY | **69** | Biotin- | SGSGTTGTMEGQY | **109** | Biotin- | SGSGGFYTVHSGS | **149** | Biotin- | SGSGERGYIRMVR |
| **30** | Biotin- | SGSGDLGLVTYTL | **70** | Biotin- | SGSGGTMEGQYMK | **110** | Biotin- | SGSGYTVHSGSEV | **150** | Biotin- | SGSGGYIRMVRNR |
| **31** | Biotin- | SGSGGLVTYTLGL | **71** | Biotin- | SGSGMEGQYMKNE | **111** | Biotin- | SGSGVHSGSEVEL | **151** | Biotin- | SGSGIRMVRNRGN |
| **32** | Biotin- | SGSGVTYTLGLNQ | **72** | Biotin- | SGSGGQYMKNERT | **112** | Biotin- | SGSGSGSEVELKN | **152** | Biotin- | SGSGMVRNRGNMC |
| **33** | Biotin- | SGSGYTLGLNQFT | **73** | Biotin- | SGSGYMKNERTSI | **113** | Biotin- | SGSGSEVELKNLV | **153** | Biotin- | SGSGRNRGNMCGI |
| **34** | Biotin- | SGSGLGLNQFTDM | **74** | Biotin- | SGSGKNERTSISF | **114** | Biotin- | SGSGVELKNLVGA | **154** | Biotin- | SGSGRGNMCGIAS |
| **35** | Biotin- | SGSGLNQFTDMTF | **75** | Biotin- | SGSGERTSISFSE | **115** | Biotin- | SGSGLKNLVGAEG | **155** | Biotin- | SGSGNMCGIASLA |
| **36** | Biotin- | SGSGQFTDMTFEE | **76** | Biotin- | SGSGTSISFSEQQ | **116** | Biotin- | SGSGNLVGAEGPA | **156** | Biotin- | SGSGCGIASLASL |
| **37** | Biotin- | SGSGTDMTFEEFK | **77** | Biotin- | SGSGISFSEQQLV | **117** | Biotin- | SGSGVGAEGPAAV | **157** | Biotin- | SGSGIASLASLPM |
| **38** | Biotin- | SGSGMTFEEFKAK | **78** | Biotin- | SGSGFSEQQLVDC | **118** | Biotin- | SGSGAEGPAAVAV | **158** | Biotin- | SGSGSLASLPMVA |
| **39** | Biotin- | SGSGFEEFKAKYL | **79** | Biotin- | SGSGEQQLVDCSR | **119** | Biotin- | SGSGGPAAVAVDV | **159** | Biotin- | SGSGASLPMVARF |
| **40** | Biotin- | SGSGEFKAKYLTE | **80** | Biotin- | SGSGQLVDCSRPW | **120** | Biotin- | SGSGAAVAVDVES | **160** | Biotin- | SGSGSLPMVARFP |

**Additional file 1: Text S1**

Formula Corrected OD (Cod): Wells which were not coated with peptides and wells coated with peptides but without plasma (blank wells) were included in triplicate in each plate. Optical density (OD) from blank wells was considered as Background (BG). The mean OD of BG for each plate was calculated and was then subtracted from the wells containing peptides and plasma in each plate, and expressed as Cod by the following formula: Cod = OD(peptide+plasma)-ODBG.

**Additional file 1: Figure S1.** Secondary and Tertiary model of FhCL1.Uniprot was used to obtain the model using the accession number Q24940 and RCSB PDB: 2O6X. The 3D diagram was created using the programme UCSF CHIMERA 1.13.1. The active site is localised at positions 132, 269, 289 (coloured purple in the centre). The pro-peptide (activation peptide, region 16-106) is indicated in yellow, the C-terminal and N-terminal are indicated in red and blue, respectively. Both figures represent the same model with a turn of 190º.


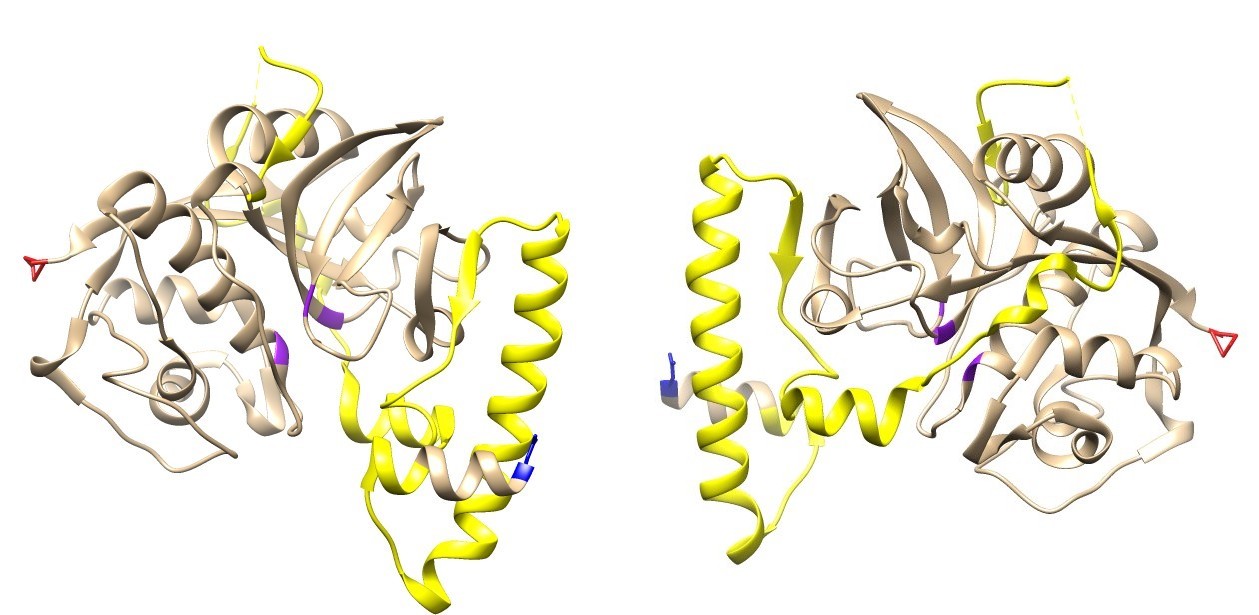


**Additional file 1: Table S3**. Level of statistical significance of peptides recognised in each group. 2-way ANOVA was performed for comparison of epitope binding. *= *P*<0.05, **= *P*<0.01; ***= *P*<0.001

| **Peptides** | | | **Group 1 (vaccinated-protected)** | | | | | **Group 2 (vaccinated -non-protected)** | | | | | **Group 3 (positive control)** | | |
| --- | --- | --- | --- | --- | --- | --- | --- | --- | --- | --- | --- | --- | --- | --- | --- |
|  |  |  | Time points | | | | | Time points | | | | | Time points | | |
| Peptide number | Amino acid  number sequence | Peptide sequence | week 0 vs  4 wav | week 0 vs 4 wai | week 0 vs  12 wai | 4 wav vs  4 wai | 4 wai vs  12 wai | week 0 vs  4 wav | week 0 vs 4 wai | week 0 vs  12 wai | 4 wav vs  4 wai | 4 wai vs  12 wai | week 0 vs  4 wai | week 0 vs  12 wai | 4 wai vs  12 wai |
| 28 | 55-63 | NLRHDLGLV | ******* | *** |  |  | *** |  |  |  |  |  |  |  |  |
| 39 | 77-84 | FEEFKAKYL | * |  |  |  |  |  |  |  |  |  |  |  |  |
| 52 | 102-110 | ANNRAVPDK | *** | *** | *** |  | ** |  |  |  |  |  |  |  |  |
| 53 | 104-112 | NRAVPDKID | *** | *** |  |  | * |  |  |  |  |  |  |  |  |
| 54 | 106-114 | AVPDKIDWR |  | *** |  | * |  |  |  |  |  |  |  |  |  |
| 55 | 108-116 | PDKIDWRES |  | *** |  | ** | ** |  |  | * |  |  |  |  |  |
| 56 | 110-118 | KIDWRESGY |  |  |  |  |  |  |  |  |  |  |  | *** | *** |
| 57 | 112-120 | DWRESGYVT |  |  |  |  |  |  |  |  |  |  |  | *** |  |
| 77 | 153-161 | ISFSEQQLV |  | * |  |  |  |  |  |  | *** |  |  |  |  |
| 102 | 203-211 | QCRYNKQLG | *** | ** |  |  |  |  | ** | *** |  | ** |  | *** | ** |
| 103 | 205-213 | RYNKQLGVA |  | * |  |  |  |  |  | ** |  |  |  |  |  |
| 133 | 265-273 | LRVNHAVLA | *** |  |  |  |  |  |  |  |  |  |  |  |  |
| 147 | 293-301 | LSWGERGYI |  |  |  |  |  |  |  | ** |  |  |  |  |  |

**Additional file 1: Table S4.** Non-specifically recognised peptides. Dynamic of corrected optical density (OD) for each peptide at different time points is given in table 3. Notice that peptides were detected in one single animal per group, only peptide 37 and 102 was commonly recognised by two animals.

| **Peptide number** | **Amino acid**  **number sequence** | **Peptide sequence** | **Nº Animal**  **Group** | **Corrected OD** | | | |
| --- | --- | --- | --- | --- | --- | --- | --- |
|  |  |  |  | **Time points** | | | |
|  |  |  |  | **week 0** | **4 wav** | **4 wai** | **12 wai** |
| 37 | 73-81 | TDMTFEEFK | 1 (G1) | 0.982 | 0.000 | 0.000 | 0.000 |
| 82 | 163-171 | CSRPWGNNG | 2 (G1) | 0.683 | 0.355 | 0.000 | 0.149 |
| 83 | 165-173 | RPWGNNGCG | 2 (G1) | 0.513 | 0.243 | 0.025 | 0.000 |
| 102 | 203-211 | QCRYNKQLG | 2 (G1) | 1.063 | 1.289 | 0.754 | 0.661 |
| 10 | 19-27 | DLWHQWKRM | 12 (G2) | 0.417 | 0.092 | 0.244 | 0.345 |
| 11 | 21-29 | WHQWKRMYN | 12 (G2) | 0.727 | 0.000 | 0.244 | 0.605 |
| 12 | 23-31 | QWKRMYNKE | 12 (G2) | 0.586 | 0.000 | 0.113 | 0.494 |
| 13 | 25-33 | KRMYNKEYN | 12 (G2) | 0.424 | 0.000 | 0.000 | 0.479 |
| 20 | 39-47 | HRRNIWEKN | 12 (G2) | 0.625 | 0.047 | 0.317 | 0.525 |
| 89 | 177-185 | MENAYQYLK | 12 (G2) | 0.374 | 0.032 | 0.000 | 0.287 |
| 90 | 179-187 | NAYQYLKQF | 12 (G2) | 0.333 | 0.051 | 0.274 | 0.264 |
| 101 | 201-209 | EGQCRYNKQ | 12 (G2) | 0.280 | 0.077 | 0.122 | 0.436 |
| 102 | 203-211 | QCRYNKQLG | 12 (G2) | 0.252 | 0.200 | 0.261 | 0.551 |
| 106 | 211-219 | GVAKVTGFY | 12 (G2) | 0.692 | 0.192 | 0.234 | 0.314 |
| 148 | 295-303 | WGERGYIRM | 12 (G2) | 0.230 | 0.219 | 0.145 | 0.265 |
| 149 | 297-305 | ERGYIRMVR | 12 (G2) | 0.482 | 0.251 | 0.131 | 0.313 |
| 150 | 299-307 | GYIRMVRNR | 12 (G2) | 0.462 | 0.369 | 0.315 | 0.359 |
| 37 | 73-81 | TDMTFEEFK | 14 (G3) | 0.553 | - | 0.219 | 0.238 |
| 55 | 108-116 | PDKIDWRES | 14 (G3) | 0.641 | - | 0.000 | 0.169 |
